# Air trapping in usual interstitial pneumonia pattern at CT: prevalence and prognosis

**DOI:** 10.1038/s41598-018-35387-3

**Published:** 2018-11-22

**Authors:** Bruno Hochhegger, Felipe Duenhas Sanches, Stephan Philip Leonhardt Altmayer, Gabriel Sartori Pacini, Matheus Zanon, Álvaro da Costa Batista Guedes, Guilherme Watte, Gustavo Meirelles, Marcelo Cardoso Barros, Edson Marchiori, Adalberto Sperb Rubin

**Affiliations:** 1 Pathology Graduate Program, Federal University of Health Sciences of Porto Alegre - R. Sarmento Leite, 245, Porto Alegre, 90050170 Brazil; 2 Medical Imaging Research Lab, LABIMED, Department of Radiology, Pavilhão Pereira Filho Hospital, Irmandade Santa Casa de Misericórdia de Porto Alegre - Av, Independência, 75, Porto Alegre, 90020160 Brazil; 3 Federal University of Health Sciences of Porto Alegre - R. Sarmento Leite, 245, Porto Alegre, 90050170 Brazil; 4 Grupo Fleury, Imaging – Av. João Pedro Cardoso, 158, São Paulo, 01333910 Brazil; 5 Department of Radiology, Pontificia Universidade Católica do Rio Grande do Sul – Av. Ipiranga 6690, Porto Alegre, RS 90619900 Brazil; 6 Department of Radiology, Federal University of Rio de Janeiro Medical School - Av. Carlos Chagas Filho, 373, Rio de Janeiro, 21941902 Brazil

## Abstract

This study was conducted to evaluate the presence of air trapping in patients with idiopathic pulmonary fibrosis (IPF) and other interstitial lung diseases (ILDs) (non-IPF), showing the radiological pattern of usual interstitial pneumonia (UIP). Retrospectively, we included 69 consecutive patients showing the typical UIP pattern on computed tomography (CT), and 15 final diagnosis of IPF with CT pattern “inconsistent with UIP” due to extensive air trapping. Air trapping at CT was assessed qualitatively by visual analysis and quantitatively by automated-software. In the quantitative analysis, significant air trapping was defined as >6% of voxels with attenuation between −950 to −856 HU on expiratory CT (expiratory air trapping index [ATIexp]) or an expiratory to inspiratory (E/I) ratio of mean lung density >0.87. The sample comprised 51 (60.7%) cases of IPF and 33 (39.3%) cases of non-IPF ILD. Most patients did not have air trapping (E/I ratio ≤0.87, n = 53, [63.1%]; ATIexp ≤6%, n = 45, [53.6%]). Air trapping in the upper lobes was the only variable distinguishing IPF from non-IPF ILD (prevalence, 3.9% vs 33.3%, p < 0.001). In conclusion, air trapping is common in patients with ILDs showing a UIP pattern on CT, as determined by qualitative and quantitative evaluation, and should not be considered to be inconsistent with UIP. On subjective visual assessment, air trapping in the upper lobes was associated with a non-IPF diagnoses.

## Introduction

Idiopathic pulmonary fibrosis (IPF) is the most common type of interstitial lung disease (ILD), with an unknown etiology and poor prognosis^1^. Commonly, IPF is the typically represented by a pattern of usual interstitial pneumonia (UIP) on both high-resolution computed tomography (HRCT) and histopathological examinations^1,2^. However, other ILDs can also present a UIP pattern on HRCT, such as chronic hypersensitivity pneumonitis (CHP) and connective tissue diseases (CTDs)^1–4^. The management and prognosis of these other ILDs are completely different from those for IPF, so; thus, diagnosis must be precise^1–4^. According to the 2011 ATS/ERS/JRS/ALAT guidelines^1^, a UIP pattern on HRCT in the absence of any identifiable cause of another ILDs is sufficient for the diagnosis of IPF, and surgical lung biopsy (SLB) is not necessary to confirm the diagnosis in such cases. On the other hand, the presence of extensive (i.e. in three or more lobes) air trapping on HRCT is currently considered to be a finding “inconsistent with UIP”, and SLB may be necessary in such cases^1^. However, SLB is associated with substantial hospitalization, and mortality rates from 2% to 7.1% in patients with ILD^5^.

Up to 31% of patients with air trapping have some form of ILD, and about 4% of these correspond to IPF^6^. In previous series, the prevalence of air trapping in patients with IPF ranged from 12.6% to 35%^7–9^. Yagihashi *et al*. found that 21.3% of cases with a definite pathological UIP pattern presented extensive bilateral air trapping^9^. Hence, air trapping is a common finding in IPF, and current guidelines stating that this CT finding is inconsistent with the diagnosis of IPF are likely too restrictive.

The primary aim of this study was to evaluate CT finding of air trapping with the in patients with IPF and those with other ILDs. A secondary aim was to examine the utility of different quantitative measurements of air trapping as CT biomarkers for the risk of death or lung transplantation in patients with IPF.

## Methods

### Participants

With the approval of the institutional review board of Santa Casa de Misericórdia de Porto Alegre Hospital and waiver of the requirement for patient consent, we retrospectively identified 101 consecutive patients who presented with the UIP pattern on chest CT between July 2012 and December 2017, regardless of the presence of air trapping on CT. Patients were identified in our weekly multidisciplinary team meetings, with discussion of all cases according to the 2011 ATS/ERS/JRS/ALAT guidelines^1^. Clinical data recorded in patients’ medical records, such as possible exposures, presence of CTDs, autoimmune features, and follow-up data, were reviewed. All imaging exams and histological samples were revised. This study was conducted in accordance with the ethical standards of the institutional research committee and with the 1964 Helsinki declaration and its later amendments.

Inclusion criteria were a UIP pattern at chest CT defined as the presence of all the following four features: subpleural and basal predominance; reticular abnormality; honeycombing with or without traction bronchiectasis; and absence of features listed as inconsistent with UIP pattern^1^. Additionally, we also included all patients (n = 15) showing a definite UIP pattern on inspiratory CT, and extensive (i.e., in three or more lobes) air trapping, despite this feature’s characterization as inconsistent with UIP^1^. Patients were excluded due to lack of follow-up with a pulmonologist after the initial CT scan or inadequacy of medical recording (n = 7); and initial CT findings inconsistent with the UIP pattern due to extensive air trapping and at least one other incompatible feature (n = 10).

Cases showing the UIP pattern on CT in the absence of any identifiable cause of another ILDs were diagnosed as IPF^1^. Patients with typical UIP pattern and clinical features suggestive of a non-IPF etiology (e.g., history of antigen exposure, clinical findings of CTD) underwent SLB for definitive diagnosis. For these cases, final diagnoses was established by multidisciplinary team consensus based on all clinical, imaging, and pathology findings. Patients were divided into IPF and non-IPF groups according to final diagnoses.

### CT protocols

All subjects underwent a paired inspiratory and expiratory chest CT with 16 × 1.25 mm collimation (LightSpeed 16 Slice Pro, General Electric Healthcare Technologies, Waukesha, WI, USA). We followed the optimal CT protocol for evaluation of ILDs recommended by the ATS/ERS/JRS/ALAT guidelines^1^. Scans were performed caudocranially using a helical acquisition. Images were reconstructed with a slice thickness and interval of 1.0 mm to achieve near-isotropic voxels. Images were acquired using the following parameters: tube voltage, 120 kV; tube current for inspiratory image, 200 mAs; tube current for expiratory images, 50 mAs; pitch, 1.375. A standard reconstruction kernel was used to achieve medium-smooth images. Reconstructions were obtained in the sagittal and coronal planes. A data matrix of 512 × 512 and a field of view of 35–45 cm were used. No CT dose modulation or intravenous contrast agent was used for this study.

### Imaging analysis

All examinations were analyzed in an Advantage Workstation (Advantage Workstation 4.6, General Electric Healthcare Technologies, Waukesha, WI, USA) from one PACS system. Inspiratory and expiratory CT images were evaluated using software designed for the assessment of segmented images from the chest wall, mediastinum, diaphragm, and airways. Automated segmentation of the right and left lungs from the chest wall and mediastinum was performed. Two chest radiologists, each with more than 8 years of experience, independently assessed the CT images blinded to the clinical information to visually qualify the presence of lobar air trapping. Images were analyzed according to criteria defined in the ATS/ERS 2011 guidelines in the Fleischner Society’s *Glossary of Terms*^1,10^. The total lung volume and attenuation of all voxels included in the lung segmentation were quantified. The expiratory air-trapping index (ATIexp) was calculated as the percentage of lung voxels with attenuation of −950 to −856HU on expiratory CT images, to exclude emphysema areas^11^. Mean lung density (MLD) histograms were created for expiratory and inspiratory acquisitions for each subject, and the expiration and inspiration (E/I) ratio of MLD was calculated^12,13^. Air trapping was defined as ATIexp > 6% or E/I ratio > 0.87. These cutoff values were determined in previous studies on COPD and asthma, and we hypothesized that they could also be applied in ILDs^13,14^. The presence and extent of air trapping were scored visually in consensus by two radiologists. In cases of discrepancy, a third radiologist with more than 15 years of experience was consulted. Air trapping was assessed according to the lobes involved, defined as sharply demarcated areas of lung tissue with lesser than normal increase in attenuation and lack of volume reduction after expiration in more than five lobules^10,15^.

### Statistical analysis

Data was presented as frequency and percentage or mean ± standard deviation (SD). Shapiro-Wilk test was used to test for normality. Continuous variables were compared using the independent Student’s *t*-test. We assessed the association between categorical variables with the Chi-Square test. Kaplan-Meier curves compared with log-rank tests were used for cumulative survival analysis. Survival analysis was performed using Cox proportional hazard regression models: (i) Events were defined as the time to death or unilateral pulmonary transplantation; (ii) censored data was used when the event did not occur at the end of the follow-up period. A p-value < 0.05 level was considered significant. Data were analyzed using Stata software, version 13 (StataCorp, College Station, TX, USA).

## Results

### Study subjects

In total, 84 consecutive patients were included in the analysis. Baseline characteristics of the study subjects are shown in Table 1. Most patients were male (n = 46, 54.7%) and smokers (n = 56, 66.6%), and the mean patient age was 66.7 ± 11.5 years. Mean follow-up of the sample was 2.2 ± 1.3 years. Most did not have air trapping (E/I ratio ≤ 0.87, n = 53, [63.1%]; ATIexp ≤ 6%, n = 45 [53.6%]). IPF was diagnosed in 51 (60.7%) cases, by SLB (n = 10) and by multidisciplinary team consensus based on clinical findings and presence of the UIP pattern on CT (n = 41). In the non-IPF group (n = 33, [39.3%]), diagnoses were CHP (n = 12), rheumatoid arthritis (n = 9), CTDs (n = 6), microscopic polyangiitis (n = 3), granulomatosis with polyangiitis (n = 1), pneumoconiosis (n = 1), and amiodarone pulmonary toxicity (n = 1).

**Table 1:** Demographic characteristics (N = 84).

Parameter	Value
Mean age ± SD (y)	66.7 ± 11.5
Patient sex, no. (%)	
Male	46 (54.7)
Female	38 (45.3)
Smokers, no. (%)	56 (66.6)
Etiology, no. (%)	
Idiopathic pulmonary fibrosis	51 (60.7)
CHP	12 (14.0)
Rheumatoid arthritis	9 (10.7)
CTD	6 (7.1)
Others	6 (7.1)
Death or lung transplantation, no. (%)	29 (34.5)
Quantitative CT measurements	
Mean MLDexp ± SD (HU)	−617.3 ± 74.5
Mean MLDinsp ± SD (HU)	−732.9 ± 56.7
E/I-ratio > 0.87, no. (%)	31 (36.9)
ATIexp > 6.0, no. (%)	39 (46.4)

Note. - ATIexp = air trapping index expiratory, CHP = chronic hypersensitivity pneumonitis, CT = computed tomography, CTD = connective tissue disease, E/I-ratio = expiratory per inspiratory ratio, MLDexp = mean lung density in expiration, MLDinsp = mean lung density in inspiration, SD = standard deviation.

### Air trapping analyses

Table 2 shows differences between IPF and the non-IPF cases revealed by the use of different criteria to define air trapping. The presence of air trapping in upper lobes was the only variable that differed significantly between groups (IPF, 2/51, [3.9%]; non-IPF, 11/33, [33.3%]; p < 0.001). No difference was observed in the prevalence of extensive air trapping (p = 0.764), ATIexp > 6% (p = 0.761), or E/I ratio > 0.87 (p = 0.313). The mean ATIexp was 9.47 (95% confidence interval [CI]: 6.66–12.29) in the IPF group and 5.83 (95% CI: 3.59–8.06) in the non-IPF group. Mean E/I ratios were similar in both groups (IPF, 0.84 [95% CI, 0.82–0.87]; non-IPF, 0.84 [95% CI, 0.81–0.88]).

**Table 2:** Qualitative and quantitative assessment of air trapping.

	IPF (n = 51)	non-IPF (n = 33)	*p*-value
Air Trapping, no. (%)			
in the superior lobe	2 (3.9)	11 (33.3)	<0.001
by ATIexp	23 (45.1)	16 (48.5)	0.761
by E-I/ratio	21 (41.2)	10 (30.3)	0.313

Note. - IPF, idiopathic pulmonary fibrosis; ATIexp, air-trapping index on expiration; E-I/ratio, expiratory to inspiratory ratio of mean lung density.

**Figure 1:**
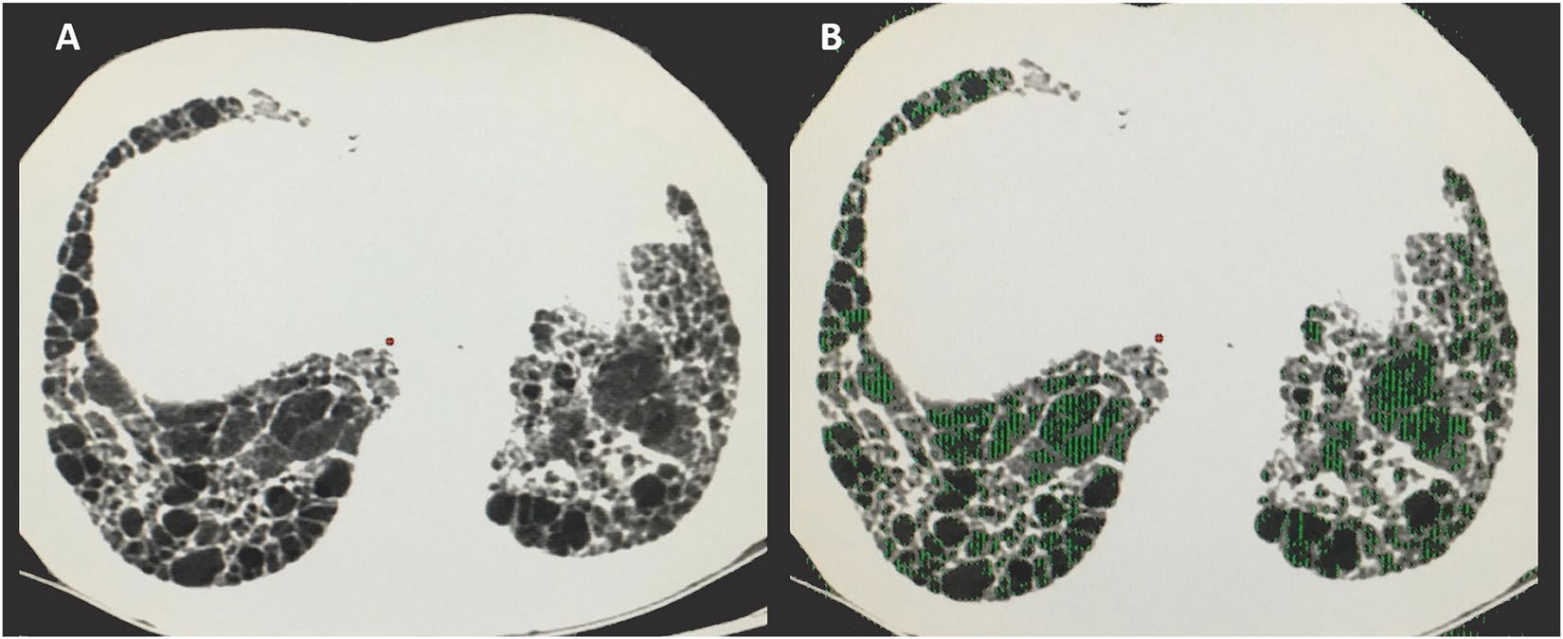
Expiratory CT from a subject with IPF associated with air trapping. Images from an 83-year-old patient diagnosed with IPF. (**A**) Axial chest CT scan demonstrates extensive areas of honeycombing in the cortical of the inferior lobes, suggestive of UIP pattern. (**B**) Same CT slice demonstrating the air trapping regions colored in green (ATIexp = 31.6%, E/I-ratio = 0.91).

**Figure 2:**
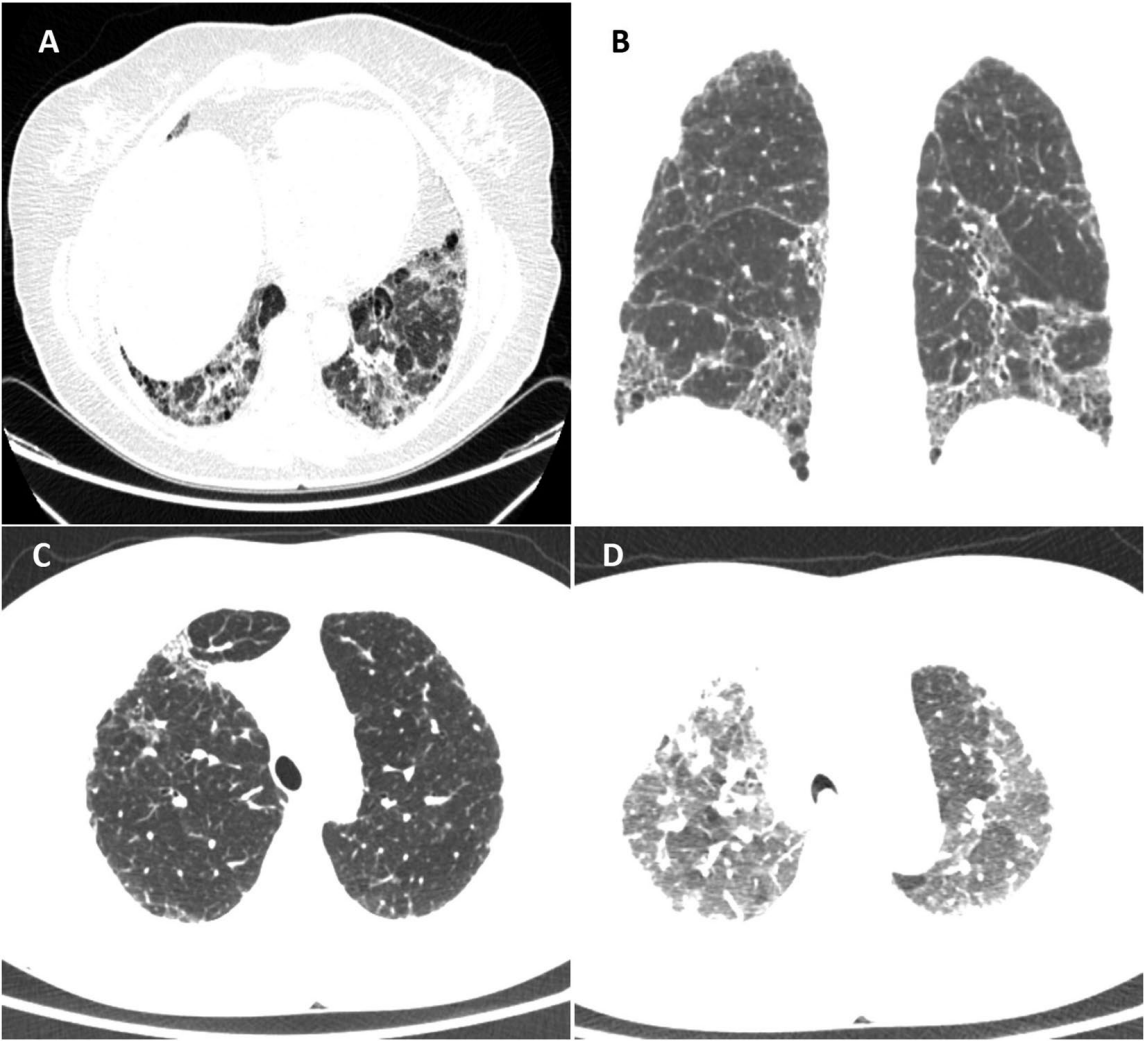
Areas of air trapping in the upper lobe on the CT of a patient with IPF. Images from a 65-year-old patient diagnosed with IPF. (**A**) Axial chest CT demonstrates areas of septal thickening and honeycombing in the lower lobes. (**B**) Predominance of a craniocaudal gradient of septal thickening, bronchiectasis, and honeycombing. (**C**) Inspiratory axial slice of the upper lobes for comparison - (**D**) Expiratory slice demonstrating diffuse air trapping in both upper lobes.

Figure 1 demonstrates the quantitative assessment of areas of air trapping in a patient with IPF. Figure 2 illustrates a case of a patient that presented an inconsistent with UIP pattern at CT due to extensive air trapping, but who had a diagnosis of IPF based on a UIP pattern in histopathology and multidisciplinary team consensus.

### Survival analysis by UIP etiology and air-trapping

**Figure 3:**
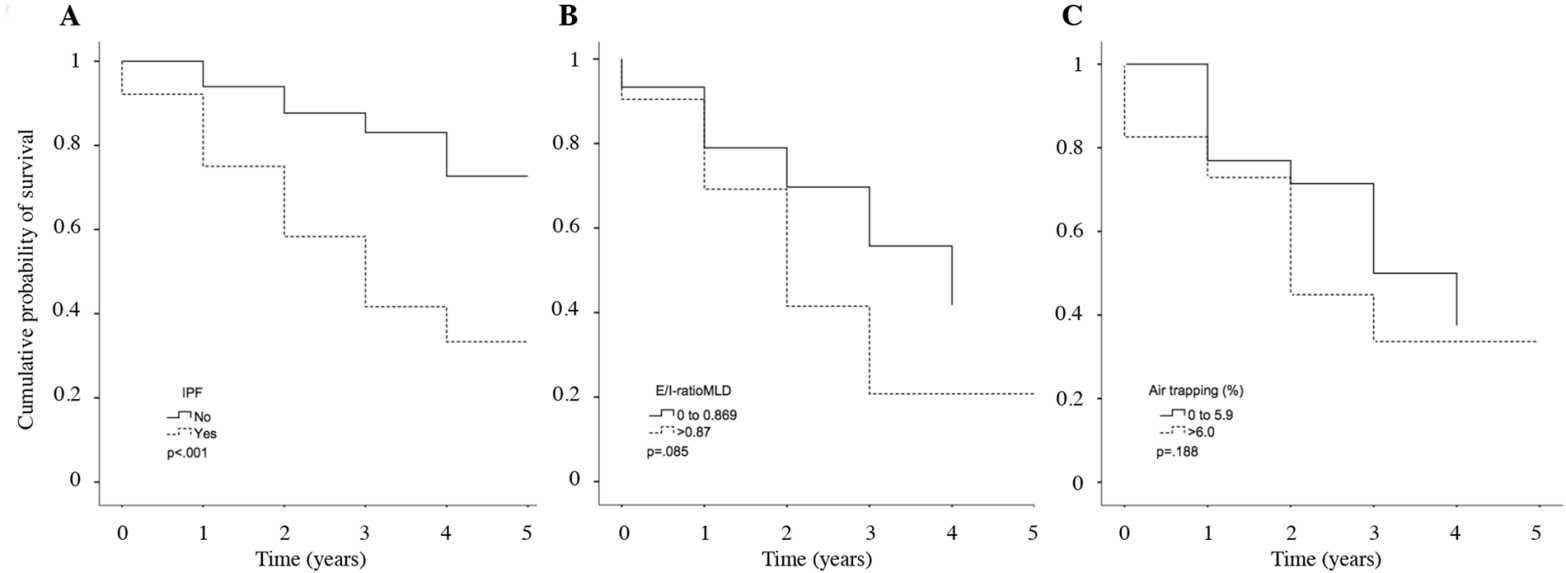
Kaplan-Meier survival analysis according to the etiology, and the impact of air trapping in patients with IPF.

During the follow-up period, 26 patients died and three underwent unilateral lung transplantation. The mean survival time was significantly shorter in the IPF group than in the non-IPF group (3 [95% 2.5–3.5] vs 4.2 [95% 3.6–4.7] years; p < 0.05). Event-free survival at 1, 3, and 5 years were also significantly lower for patients with IPF than those with other ILDs (81%, 45%, and 36% vs. 93%, 80%, and 66%, respectively; all p < 0.004) (Fig. 3A).

Among patients with IPF, we analyzed differences in the cumulative survival between patients with significant air trapping defined by quantitative CT measurements (i.e., E/I-ratio > 0.87 and ATIexp > 6%). Although these patients had slightly worse event-free survival at 1, 3, and 5 years, these differences were not significant (p = 0.085 and 0.188) (Fig. 3).

## Discussion

Air trapping was a common finding in patients with ILD, present in up to 45.1% of patients with IPF in this sample. Moreover, the degree of air trapping did not differ between patients with IPF and those with UIP of known etiology according to either index used in this study. However, the finding of air trapping in the superior lobe is rare present in patients with IPF, and thus may be highly suggestive of an alternative diagnosis when present in patients with a UIP pattern.

The differential clinical diagnosis of radiological UIP is challenging, as it requires distinction of several conditions, including IPF^1–4^. Imaging findings may be similar to those for non-specific interstitial pneumonia, CHP, or sarcoidosis, which may lead to initial difficulty of diagnosis, especially when they are the only parameters considered^16^. The most recent (2011) ATS/ERS guidelines emphasized the importance of multidisciplinary discussion among radiologists, pulmonologists and pathologists for the diagnosis of IPF, to increase interobserver agreement^1,17^. In this study, cases showing the typical UIP pattern on CT in the absence of any identifiable cause of another ILD were diagnosed as IPF to avoid an unnecessary performance of invasive procedure^18^. However, when CT demonstrated air trapping in more than three lobes, patients underwent SLB to establish the final diagnosis. The greater mortality rate in patients with IPF relative to those with other types of UIP supports the accuracy of diagnoses in our study population.

Small airway obstruction with subsequent air trapping is a common finding in several pulmonary conditions, such as obstructive diseases, bronchiectasis, and ILD^6,19^. Although no cutoff value clearly defining normality has been established, current recommended for the quantitative measurement of air trapping state that the is the E/I ratio is the best method^20^. In our study, we have used the cutoff of 0.87, which was found to be the optimal cutoff to depict air trapping in previous studies on COPD an asthma^20^. In general, the greater the ratio, the greater the air trapping^20^. Among the various forms of ILD, CHP is characterized by air trapping as a main tomographic finding^16^. In certain types of CHP, HRCT may demonstrate the pattern of UIP, associated with a reduction in pulmonary volume, and the presence of linear and reticular opacities and honeycombing^21^. The presence of centrilobular nodules, lobular areas with decrease attenuation, and predominance in the superior regions of lung tend to differentiate CHP from IPF^8,22^. In our cohort, 11 of the 13 patients with air trapping in the superior lobes were diagnosed with CHP, and the other two patients were diagnosed with IPF.

Many biopsy-proven cases of UIP present on HRCT as a “probable UIP” pattern or even in the presence of inconsistent findings with UIP^16,23^. SLB may not have been necessary in some of these cases, and its avoidance spares patients from associated comorbidities. Some areas of hypoattenuated lung appearing on expiratory imaging, considered to represent air trapping in visual or quantitative analysis, could actually be segments of severely fibrotic non-compliant lung that fail to recoil during expiration. On the other hand, our study findings support consideration of the presence of air trapping in the superior lobes as inconsistent with the UIP pattern and warranting SLB. Moreover, software-assisted quantification of air trapping using the cutoffs employed in this study did not distinguish IPF from other causes of radiological UIP. Howerver, patients with IPF and air trapping, as determined by the E/I ratio, showed a trend toward worse prognosis during follow-up.

Our study has some limitations. First, it likely involved selection bias, as our institution is a local reference center for the diagnosis and management of ILD, especially IPF, which may have increased the prevalence of these diseases relative to other causes of UIP in our sample. For this reason, the sample may not be representative of the overall prevalence of IPF in the general population, although the clinical outcome of our patients resembles those reported previously^24^. Second, the study sample was small (n = 84). Studies with larger samples should be conducted to corroborate our results. Given the limitations inherent to the retrospective nature of this study, some clinical parameters (e.g.; medication use, comorbidities, functional status) for patients at the time of the CT examination could not be retrieved. Third, the quantification of air trapping and distinction of this condition from cystic areas of honeycombing or emphysema can be challenging; for this reason, we did not include areas of attenuation below −950 HU, and we performed visual analysis of the areas of air trapping delimitated by the software when necessary. Lastly, the literature contains no data substantiating the quantitative assessment of air trapping in subjects with IPF. Further studies are need to establish more accurate cutoffs values for air trapping in this population.

In conclusion, air trapping is common in patients with ILDs showing the UIP pattern on CT, as determined by qualitative and quantitative evaluation, and it should not be considered to be inconsistent with UIP. On the subjective visual assessment, air trapping in the upper lobes was associated with non-IPF diagnoses.

## References

[1] Raghu G (2011). An Official ATS/ERS/JRS/ALAT Statement: Idiopathic Pulmonary Fibrosis: Evidence-based Guidelines for Diagnosis and Management. Am J Respir Crit Care Med.

[2] Ley B, Collard HR, King TE (2011). Clinical Course and Prediction of Survival in Idiopathic Pulmonary Fibrosis. Am J Respir Crit Care Med.

[3] Morell F (2013). Chronic hypersensitivity pneumonitis in patients diagnosed with idiopathic pulmonary fibrosis: a prospective case-cohort study. Lancet Respir Med.

[4] Wuyts WA (2014). Differential diagnosis of usual interstitial pneumonia: when is it truly idiopathic?. Eur Respir Rev.

[5] Lettieri CJ, Veerappan GR, Helman DL, Mulligan CR, Shorr AF (2005). Outcomes and safety of surgical lung biopsy for interstitial lung disease. Chest..

[6] Miller WT, Chatzkel J, Hewitt MG (2014). Expiratory Air Trapping on Thoracic Computed Tomography. A Diagnostic Subclassification. Ann Am Thorac Soc.

[7] Chung JH (2018). CT-Pathologic Correlation of Major Types of Pulmonary Fibrosis: Insights for Revisions to Current Guidelines. Am J Roentgenol.

[8] Silva CIS (2008). Chronic hypersensitivity pneumonitis: differentiation from idiopathic pulmonary fibrosis and nonspecific interstitial pneumonia by using thin-section CT. Radiology.

[9] Yagihashi K (2016). Radiologic–pathologic discordance in biopsy-proven usual interstitial pneumonia. European Respiratory Journal.

[10] Hansell DM (2008). Fleischner Society: Glossary of terms for thoracic imaging. Radiology.

[11] Busacker A (2009). A Multivariate Analysis of Risk Factors for the Air-Trapping Asthmatic Phenotype as Measured by Quantitative CT Analysis. Chest.

[12] Eda S (1997). The relations between expiratory chest CT using helical CT and pulmonary function tests in emphysema. Am J Respir Crit Care Med.

[13] Mets OM (2012). Variation in quantitative CT air trapping in heavy smokers on repeat CT examinations. Eur Radiol.

[14] Lee KW (2000). Correlation of aging and smoking with air trapping at thin-section CT of the lung in asymptomatic subjects. Radiology.

[15] Sahin H (2007). Chronic hypersensitivity pneumonitis: CT features comparison with pathologic evidence of fibrosis and survival. Radiology..

[16] Sverzellati N (2010). Biopsy-proved idiopathic pulmonary fibrosis: spectrum of nondiagnostic thin-section CT diagnoses. Radiology.

[17] Flaherty KR (2004). Idiopathic interstitial pneumonia: what is the effect of a multidisciplinary approach to diagnosis?. Am J Respir Crit Care Med.

[18] Mikolasch TA, Garthwaite HS, Porter JC (2017). Update in diagnosis and management of interstitial lung disease. Clin Med.

[19] Arakawa H, Webb WR (1998). Air trapping on expiratory high-resolution CT scans in the absence of inspiratory scan abnormalities: correlation with pulmonary function tests and differential diagnosis. Am J Roentgenol.

[20] Mets OM (2012). Early Identification of Small Airways Disease on Lung Cancer Screening CT: Comparison of Current Air Trapping Measures. Lung.

[21] Chung MH (2001). Mixed infiltrative and obstructive disease on high-resolution CT: differential diagnosis and functional correlates in a consecutive series. J Thorac Imaging.

[22] Lynch DA (1995). Can CT distinguish hypersensitivity pneumonitis from idiopathic pulmonary fibrosis?. Am J Roentgenol.

[23] Chung JH (2015). CT Scan Findings of Probable Usual Interstitial Pneumonitis Have a High Predictive Value for Histologic Usual Interstitial Pneumonitis. Chest.

[24] European, Respiratory Society, and American Thoracic Society American Thoracic Society/European Respiratory Society International Multidisciplinary Consensus Classification of the Idiopathic Interstitial Pneumonias. This joint statement of the American Thoracic Society (ATS), and the European Respiratory Society (ERS) was adopted by the ATS board of directors, June 2001 and by the ERS Executive Committee, June 2001. *Am J Respir Crit Care Med* **165**, 277 (2002). 10.1164/ajrccm.165.2.ats01 11790668

